# In the March 2014 issue

**DOI:** 10.1590/S1980-57642014DN81000001

**Published:** 2014

**Authors:** Ricardo Nitrini

**Affiliations:** Editor-in-Chief

In this issue we are publishing two reviews, eight original papers, two history notes and two case reports.

**Mattos-Parente et al.** reviewed the literature to verify whether patients with Parkinson's disease (PD) have more difficulties in the semantic processing of action concepts (action verbs) than motionless objects, supporting the Embodied Cognition model. The authors concluded that much more work must be carried out to understand how the brain represents the complex semantics of the verbs and to ascertain which semantic strategies can help patients with specific difficulties in verb processing.

**Ianof et al.** performed a literature review on sport-related concussions and concluded that concussions are seldom recognized and frequently unreported. Mismanaging of concussion can cause persistent post-concussion syndrome and/or second-impact syndrome, making concussion recognition and proper medical supervision of vital importance.

**Rocha et al.** investigated the accuracy of the Brazilian version of Addenbrooke's Cognitive Examination-Revised (ACE-R) in the cognitive assessment of patients with Parkinson's disease (PD) with heterogeneous educational background, using the diagnostic procedures of the Movement Disorder Society as a reference. ACE-R was considered a valid tool for dementia evaluation in PD, with high sensitivity and specificity.

**Campanholo et al.** recruited 978 healthy adult Brazilian subjects to obtain normative data and study the interference of age, gender and education on the trail making test (TMT) and Stroop test (ST). Gender did not exert a major impact on performance in these tests, whereas age and education had a clear influence and should therefore be taken into account in the stratification of normative samples.

**Haase et al.** investigated the neuropsychological performance of Brazilian children and adolescents with human immunodeficiency virus (HIV) infection, finding lower performance especially on motor and cognitive processing speed and executive function. The neuropsychological impairments showed a trend towards greater severity in participants with more advanced stages of the disease, however they did not correlate with the Centers for Disease Control and Prevention (CDC) stage of the HIV infection.

**Cieto et al.** performed a systematic review of the literature on public health policies for dementia care in the world and Brazil. The review included 45 studies from five continents. Developed countries tend to be more advanced in health care for dementia, although a few developing countries also promote health care plans. In Brazil, there are government healthcare programs and policies focusing on the elderly population, but the absence of effective collaboration among the federal, state and municipal policy makers is a hindrance for dementia care.

**Beber and Chaves** carried out a systematic review on the mechanism and clinical application of the action fluency (AF) task (i.e.: the ability to generate verbs in a given period of time) and the action naming (AN) task (i.e.: the ability to name a limited number of verbs graphically represented), which are verb generation tasks. The authors included 40 studies in this review and were able to conclude that AF and AN involve different brain processes, may differentiate Primary Progressive Aphasias from other conditions, and that AF is related to executive function.

**Gindri et al.** performed a systematic review of the methods used in the rehabilitation of discourse following acquired brain injury. The authors were able to retrieve few studies, and only one reported improvement following participation in a discourse rehabilitation program. Their review pointed to the need for further research and publication in discourse rehabilitation among populations with acquired brain injury.

**Miranda and Brucki** carried out a systematic review on the epidemiological, clinical and treatment aspects of epilepsy associated with Alzheimer's disease (AD). Their most relevant findings were that epilepsy is more frequent among AD patients, and neuronal hyperexcitability due to beta-amyloid peptide or phosphorylated tau protein accumulation, as well as structural changes in cortical and hippocampal regions, may increase the likelihood of epilepsy. Also, the new generation of antiepileptic drugs may be the best choice for treatment of epilepsy in AD.

**Anauate et al.** investigated the performance of patients with frontotemporal lobar degeneration (FTLD) with an artistic protocol including copying and collage activities. FTLD patients had visuospatial impairment and tended to produce drawings with a simpler composition, while both copy and collage activities were poorly and rapidly executed.

**Engelhardt** described in a history note the remarkable hypotheses of Brown-Séquard on brain organization for mental and physical functions. Ahead of his time, Brown-Séquard proposed a "network of anastomosing cells", dynamically submitted to "dynamogenic and inhibitory activities" to explain the functional organization of the brain.

**Nitrini** revisited in a history note one of the first clinical and pathological case reports of dementia in amyotrophic lateral sclerosis. The report was published in a Brazilian journal, by Tretiakoff and Amorim, in 1924.

**Simabukuro et al.** reported a case of anti-NMDA receptor encephalitis not associated with tumor that failed to show significant improvement until plasmapheresis was initiated on the 45^th^ day of evolution. After 19 sessions of plasmapheresis the patient made a substantial recovery.

**Menezes et al.** reported a case of transient global amnesia with hyperintense sign on diffusion-weighted magnetic resonance imaging in the anterior portion of the cingulate gyrus. The authors emphasized that, to their knowledge, this was an unprecedented finding.

**Ricardo Nitrini** Editor-in-Chief

